# Supplementary material for: Loss of gut microbial diversity in the cultured, agastric fish, Mexican pike silverside (Chirostoma estor: Atherinopsidae)
Source: PeerJ. 2022 Mar 7;10:e13052. doi: 10.7717/peerj.13052 (PMC8908885; doi:10.7717/peerj.13052)
Supplement: Supplemental Information 1 — Minimum, maximum, average, and standard deviation values of body or gut weight and length of Chirostoma estor harvested from each environment including Lake Patzcuaro, Intensive Culture, and Extensive Culture. [file peerj-10-13052-s001.docx]

|  |  |  |  |  |  |
| --- | --- | --- | --- | --- | --- |
| **Sample** | **Environment** | **Weight fish (g)** | **Length fish (cm)** | **Weight gut (g)** | **Length gut (cm)** |
| 1_LP | Lake Patzcuaro | 28.5 | 15.5 | 0.7 | 7.6 |
| 2_LP | Lake Patzcuaro | 27.2 | 15.6 | 0.6 | 7.5 |
| 3_LP | Lake Patzcuaro | 25.2 | 14.5 | 1.7 | 6.5 |
| 4_LP | Lake Patzcuaro | 11 | 12 | 0.2 | 4 |
| 5_LP | Lake Patzcuaro | 23.5 | 14.3 | 0.7 | 8.5 |
| 6_LP | Lake Patzcuaro | 13.3 | 16.2 | 0.7 | 8.5 |
| 7_LP | Lake Patzcuaro | 12.5 | 11.3 | 0.3 | 4.5 |
| 8_LP | Lake Patzcuaro | 33.3 | 17.3 | 0.8 | 5.8 |
| 9_LP | Lake Patzcuaro | 15.5 | 12 | 0.2 | 8 |
| 10_LP | Lake Patzcuaro | 28 | 15.8 | 0.6 | 7.7 |
| 11_LP | Lake Patzcuaro | 20.7 | 14.5 | 1.9 | 9 |
| 12_LP | Lake Patzcuaro | 23.4 | 16 | 1 | 5 |
| 13_LP | Lake Patzcuaro | 24.4 | 15 | 2.2 | 10 |
| 1_C | Intensive Culture | 52 | 15.5 | 1 | 9 |
| 2_C | Intensive Culture | 37 | 15.5 | 1 | 5 |
| 3_C | Intensive Culture | 30 | 15 | 1.4 | 3.5 |
| 4_C | Intensive Culture | 35 | 15.8 | 0.64 | 4.5 |
| 5_C | Intensive Culture | 31 | 15.3 | 1.5 | 8 |
| 6_C | Intensive Culture | 42 | 17.2 | 0.7 | 5.5 |
| 7_C | Intensive Culture | 32 | 16 | 1.5 | 8 |
| 8_C | Intensive Culture | 33 | 15.3 | 1 | 6.5 |
| 9_C | Intensive Culture | 31 | 15.3 | 0.9 | 8.3 |
| 10_C | Intensive Culture | 43 | 17 | 0.9 | 6.2 |
| 11_C | Intensive Culture | 35 | 15.5 | 1.1 | 7.8 |
| 12_C | Intensive Culture | 37 | 16.3 | 0.5 | 5.2 |
| 13_C | Intensive Culture | 45 | 17.5 | 2.2 | 11 |
| 14_C | Intensive Culture | 36.9 | 15.8 | 0.5 | 5.8 |
| 15_C | Intensive Culture | 40 | 16 | 1 | 10 |
| 16_C | Intensive Culture | 24.8 | 14.5 | 0.6 | 5.8 |
| 17_C | Intensive Culture | 51.2 | 18.3 | 2.1 | 12 |
| 18_C | Intensive Culture | 36.1 | 15.5 | 1.2 | 8.2 |
| 19_C | Intensive Culture | 27.5 | 15 | 1.2 | 8 |
| 20_C | Intensive Culture | 36.2 | 14.5 | 0.8 | 6.8 |
| 1_E | Extensive Culture | 20.5 | 14.5 | 0.4 | 6.5 |
| 2_E | Extensive Culture | 18.1 | 14 | 0.5 | 6 |
| 3_E | Extensive Culture | 31.7 | 17 | 0.8 | 10 |
| 4_E | Extensive Culture | 28.6 | 16.5 | 0.8 | 7.4 |
| 5_E | Extensive Culture | 19.2 | 13.5 | 0.5 | 10.3 |
| 6_E | Extensive Culture | 20.4 | 14.8 | 0.3 | 4.5 |
| 7_E | Extensive Culture | 21 | 14.5 | 0.7 | 8 |
| 8_E | Extensive Culture | 21 | 14.9 | 0.4 | 7.8 |
| 9_E | Extensive Culture | 17.4 | 14 | 0.5 | 9 |
| 10_E | Extensive Culture | 19 | 14.2 | 0.5 | 8.2 |
| 11_E | Extensive Culture | 18.1 | 14 | 0.3 | 10 |
| 12_E | Extensive Culture | 21 | 14.5 | 0.3 | 7.2 |
| 13_E | Extensive Culture | 20.4 | 14.3 | 0.6 | 9.5 |
| 14_E | Extensive Culture | 21.6 | 14.6 | 0.4 | 8.4 |
| 15_E | Extensive Culture | 21.9 | 15 | 0.5 | 9.5 |
| 16_E | Extensive Culture | 16.9 | 13.4 | 0.4 | 6.4 |
| 17_E | Extensive Culture | 18.9 | 14.5 | 0.6 | 9 |
| 18_E | Extensive Culture | 14 | 13 | 0.4 | 7.5 |
| 19_E | Extensive Culture | 16.7 | 13 | 0.4 | 11 |
| Total environments | Minimun | 11.00 | 11.30 | 0.20 | 3.50 |
|  | Maxium | 52.00 | 18.30 | 2.20 | 12.00 |
|  | Average | 27.09 | 15.02 | 0.82 | 7.58 |
|  | Standard Deviation | 9.83 | 1.39 | 0.51 | 1.95 |
| Lake Patzcuaro | Minimun | 11.00 | 11.30 | 0.20 | 4.00 |
|  | Maxium | 33.30 | 17.30 | 2.20 | 10.00 |
|  | Average | 22.04 | 14.62 | 0.89 | 7.12 |
|  | Standard Deviation | 6.98 | 1.82 | 0.64 | 1.83 |
| Intensive Culture | Minimun | 24.80 | 14.50 | 0.50 | 3.50 |
|  | Maxium | 52.00 | 18.30 | 2.20 | 12.00 |
|  | Average | 36.79 | 15.84 | 1.09 | 7.26 |
|  | Standard Deviation | 7.15 | 0.99 | 0.47 | 2.19 |
| Extensive Culture | Minimun | 14.00 | 13.00 | 0.30 | 4.50 |
|  | Maxium | 31.70 | 17.00 | 0.80 | 11.00 |
|  | Average | 20.34 | 14.43 | 0.49 | 8.22 |
|  | Standard Deviation | 4.03 | 1.01 | 0.15 | 1.67 |
