# Supplementary material for: Loss of gut microbial diversity in the cultured, agastric fish, Mexican pike silverside (Chirostoma estor: Atherinopsidae)
Source: PeerJ. 2022 Mar 7;10:e13052. doi: 10.7717/peerj.13052 (PMC8908885; doi:10.7717/peerj.13052)
Supplement: Supplemental Information 2 — Total mean relative abundance (%) (first column) ±SE and mean relative abundance ±SE of the 9 most prevalent phyla and 2 Classes of Proteobacteria found in intestinal components (D, A and P) samples of C. estor of three environments (LP, C, and E). Statistical comparisons are separated by intestinal components (digesta, D; anterior intestine, A; posterior intestine, P) and environments (Lake Patzcuaro, LP; Intensive Culture, C; Extensive Culture, E). Different letters indicate a statistically significant (p ¡ 0.05) difference between taxonomic group abundances per row. [file peerj-10-13052-s002.docx]

|  |  |  |  |  |  |  |  |
| --- | --- | --- | --- | --- | --- | --- | --- |
| **Phylum** | **Total abundance (n=74)** | **Intestinal component** | | | **Environment** | | |
|  |  | **D (n=24)** | **A (n=25)** | **P (n=25)** | **LP (n=24)** | **C (n=26)** | **E (n=24)** |
| Firmicutes | 56.27 ± 4.28 | 45.88 ± 7.01 | 66.09 ± 7.50 | 56.42 ± 7.41 | 44.92 ± 7.11 | 68.18 ± 6.61 | 54.72 ± 8.04 |
| Gammaproteobacteria | 24.35 ± 3.16 | 29.25 ± 6.38 | 20.96 ± 4.80 | 23.03 ± 5.26 | 23.22 ± 4.38 | 19.88 ± 5.27 | 30.32 ± 6.56 |
| Cyanobacteria | 4.69 ± 1.57 | 8.10 ± 4.03 | 1.49 ± 0.74 | 4.61 ± 2.39 | 5.53 ± 2.07 **b** | 6.62 ± 3.82 **a** | 1.75 ± 1.41 **c** |
| Alphaproteobacteria | 4.33 ± 1.25 | 2.88 ± 0.97 | 5.09 ± 2.22 | 4.98 ± 2.85 | 7.11 ± 2.97 **a** | 3.08 ± 1.77 **b** | 2.92 ± 1.51 **b** |
| Actinobacteriota | 3.51 ± 1.35 | 5.74 ± 3.77 | 1.65 ± 0.76 | 3.24 ± 1.54 | 3.99 ± 1.03 **b** | 0.97 ± 0.49 **ac** | 5.80 ± 3.99 **a** |
| Bacteroidota | 1.78 ± 0.37 | 2.24 ± 0.88 | 1.47 ± 0.39 | 1.66 ± 0.59 | 3.37 ± 0.92 **a** | 0.68 ± 0.27 **b** | 1.40 ± 0.47 **b** |
| Desulfobacterota | 1.43 ± 0.44 | 1.68 ± 0.77 | 1.43 ± 0.68 | 1.20 ± 0.86 | 3.40 ± 1.19 **a** | 0.01 ± 0.01 **c** | 1.00 ± 0.47 **b** |
| Fusobacteriota | 1.39 ± 1.05 | 0.34 ± 0.17 | 0.46 ± 0.43 | 3.33 ± 3.08 | 4.13 ± 3.21 **a** | 0.12 ± 0.10 **b** | 0.04 ± 0.03 **b** |
| Planctomycetota | 0.97 ± 0.40 | 2.51 ± 1.17 | 0.18 ± 0.07 | 0.27 ± 0.20 | 2.82 ± 1.16 **a** | 0.00 ± 0.00 **c** | 0.16 ± 0.05 **b** |
| Verrucomicrobiota | 0.38 ± 0.10 | 0.71 ± 0.24 **A** | 0.23 ± 0.10 **AB** | 0.21 ± 0.13 **B** | 0.73 ± 0.24 **a** | 0.02 ± 0.01 **b** | 0.42 ± 0.15 **a** |
| Acidobacteriota | 0.29 ± 0.16 | 0.18 ± 0.06 | 0.17 ± 0.07 | 0.51 ± 0.46 | 0.21 ± 0.06 **a** | 0.04 ± 0.04 **b** | 0.63 ± 0.48 **a** |
|  |  |  |  |  |  |  |  |
