# Supplementary material for: Loss of gut microbial diversity in the cultured, agastric fish, Mexican pike silverside (Chirostoma estor: Atherinopsidae)
Source: PeerJ. 2022 Mar 7;10:e13052. doi: 10.7717/peerj.13052 (PMC8908885; doi:10.7717/peerj.13052)
Supplement: Supplemental Information 3 — Observed, richness Chao and Shannon diversity mean relative abundance (%) ±SD of intestinal microbiota compared between environments (Lake Patzcuaro, LP; Intensive Culture, C; Extensive Culture, E), intestinal components (Digesta, D; Anterior intestine, A; Posterior intestine, P) and all 9 sample groups (environment by intestinal component). Digesta of Lake Patzcuaro, DLP; Digesta of Intensive Culture, DC; Digesta of Extensive Culture, DE; Anterior intestine of Lake Patzcuaro, ALP; Anterior intestine of Intensive Culture, AC; Anterior intestine of Extensive Culture, AE; Posterior intestine of Lake Patzcuaro, PLP; Posterior intestine of Intensive Culture, PC; Posterior Intestine of Extensive Culture, PE. In parenthesis the n number of samples. Different letters indicate statistical significance between groups (p <0.05). [file peerj-10-13052-s003.docx]

|  |  |  |  |
| --- | --- | --- | --- |
|  | **Observed** | **Chao** | **Shannon** |
| D (24) | 626.63 ± 429.03 **a** | 966.94 ± 589.89 **a** | 2.51 ± 1.23 **a** |
| A (25) | 702.04 ± 478.07 **a** | 1128.60 ± 692.63 **a** | 2.51 ± 1.19 **a** |
| P (25) | 543.16 ± 372.16 **a** | 919.31 ± 476.35 **a** | 2.41 ± 1.26 **a** |
| LP (24) | 703.04 ± 369.14 **a** | 1082.53 ± 533.81 **a** | 2.83 ± 0.86 **a** |
| C (26) | 508.42 ± 277.56 **b** | 913.15 ± 486.03 **a** | 1.95 ± 0.90 **b** |
| E (24) | 669.88 ± 580.51 **ab** | 1028.40 ± 743.87 **a** | 2.70 ± 1.59 **ab** |
| DLP (8) | 692.25 ± 430.77 **a** | 1012.19 ± 574.83 **a** | 3.12 ± 0.86 **a** |
| DC (8) | 448.00 ± 162.66 **a** | 784.62 ± 346.61 **a** | 1.82 ± 0.86 **b** |
| DE (8) | 739.63 ± 580.35 **a** | 1104.00 ± 794.33 **a** | 2.60 ± 1.59 **ab** |
| ALP (8) | 809.75 ± 345.41 **a** | 1287.7 ± 547.92 **a** | 2.73 ± 0.59 **a** |
| AC (9) | 677.25 ± 397.28 **a** | 1134.26 ± 670.29 **a** | 2.41 ± 1.21 **a** |
| AE (8) | 674.63 ± 680.79 **a** | 1048.97 ± 890.35 **a** | 2.54 ± 1.66 **a** |
| PLP (8) | 607.13 ± 345.25 **a** | 947.61 ± 480.67 **a** | 2.63 ± 1.10 **a** |
| PC (9) | 461.63 ± 185.08 **a** | 932.81 ± 375.98 **a** | 1.76 ± 0.56 **a** |
| PE (8) | 595.38 ± 543.18 **a** | 932.24 ± 613.90 **a** | 2.96 ± 1.69 **a** |
