# Supplementary material for: Loss of gut microbial diversity in the cultured, agastric fish, Mexican pike silverside (Chirostoma estor: Atherinopsidae)
Source: PeerJ. 2022 Mar 7;10:e13052. doi: 10.7717/peerj.13052 (PMC8908885; doi:10.7717/peerj.13052)
Supplement: Supplemental Information 4 — Analysis of similarity (ANOSIM) and Permutational multivariate analysis of variance (Adonis) of intestinal microbiota data of environments. [file peerj-10-13052-s004.docx]

| **Statistics** | **Bray-Curtis** |  |  |
| --- | --- | --- | --- |
| ANOSIM |  |  |  |
| Permutation N | 999 |  |  |
| R | 0.3119 |  |  |
| *p* (same) | **0.001** |  |  |
| Pairwise comparisons (adonis) | *p* value | R^2^ | F value |
| LP vs C | **0.001** | 0.0710 | 3.6688 |
| LP vs E | **0.001** | 0.0834 | 4.1834 |
| C vs E | **0.001** | 0.0490 | 2.4745 |
