# Supplementary material for: Loss of gut microbial diversity in the cultured, agastric fish, Mexican pike silverside (Chirostoma estor: Atherinopsidae)
Source: PeerJ. 2022 Mar 7;10:e13052. doi: 10.7717/peerj.13052 (PMC8908885; doi:10.7717/peerj.13052)
Supplement: Supplemental Information 5 — Analysis of similarity (ANOSIM) and Permutational multivariate analysis of variance (Adonis) of data of intestinal microbiota between intestinal components. [file peerj-10-13052-s005.docx]

| **Statistics** | **Bray-Curtis** |  |  |
| --- | --- | --- | --- |
| ANOSIM |  |  |  |
| Permutation N | 999 |  |  |
| R | 0.06763 |  |  |
| *p* (same) | 0.004 |  |  |
| Pairwise comparisons (adonis) | *p* value | R^2^ | F value |
| D vs A | **0.001** | 0.0454 | 2.2341 |
| D vs P | 0.061 | 0.0294 | 1.4241 |
| A vs P | 0.600 | 0.0186 | 0.9091 |
