# Supplementary material for: Loss of gut microbial diversity in the cultured, agastric fish, Mexican pike silverside (Chirostoma estor: Atherinopsidae)
Source: PeerJ. 2022 Mar 7;10:e13052. doi: 10.7717/peerj.13052 (PMC8908885; doi:10.7717/peerj.13052)
Supplement: Supplemental Information 6 — Forty-two OTUs were annotated at the genus level using the SILVA database. There were a total of core 133 OTUs found in the UpSet plot of intestinal components and environments samples (Fig. 3E). In bold are genera present in the core microbiota above the threshold of 80% occupancy (Figs. 3A–3D). [file peerj-10-13052-s006.docx]

|  |  | |  |
| --- | --- | --- | --- |
|  | **Genus present in the annotated sequences by SILVA database** | |  |
|  | *Pelomonas* | *Mesorhizobium* |  |
|  | ***Acinetobacter*** | *Methylobacterium-Methylorubrum* |  |
|  | ***Aeromonas*** | ***Mycoplasma*** |  |
|  | *Aquabacterium* | *Novosphingobium* |  |
|  | ***Bacillus*** | ***Pseudomonas*** |  |
|  | *Bradyrhizobium* | *Ralstonia* |  |
|  | *Caulobacter* | *Reyranella* |  |
|  | *Cetobacterium* | *Rhodoluna* |  |
|  | *Chloroflexus* | *Romboutsia* |  |
|  | *Citrobacter* | *Rubrivivax* |  |
|  | *Comamonas* | *Salinibacterium* |  |
|  | *Cutibacterium* | *Shewanella* |  |
|  | *Enhydrobacter* | *Shimwellia* |  |
|  | *Erwinia* | *Sphingomonas* |  |
|  | *Foliisarcina* | ***Spiroplasma*** |  |
|  | *Gemmobacter* | ***Staphylococcus*** |  |
|  | *Hydrobacter* | *Stenotrophomonas* |  |
|  | *Hyphomicrobium* | *Streptococcus* |  |
|  | *Iamia* | ***Vibrio*** |  |
|  | *Iphinoe* | *Vogesella* |  |
|  | *Massilia* | ***Weissella*** |  |
