# Supplementary figures and images for: Loss of gut microbial diversity in the cultured, agastric fish, Mexican pike silverside (Chirostoma estor: Atherinopsidae)
Source: PeerJ. 2022 Mar 7;10:e13052. doi: 10.7717/peerj.13052 (PMC8908885; doi:10.7717/peerj.13052)

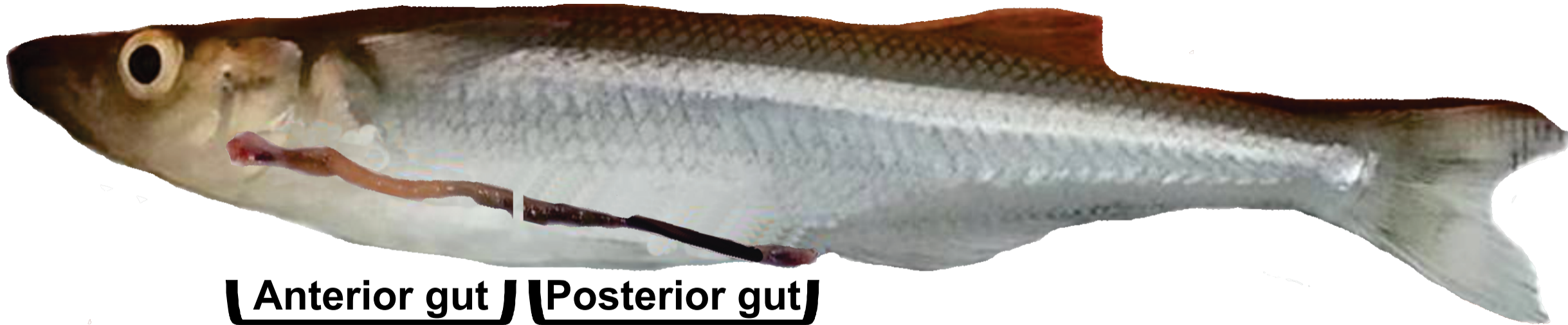

**[Anterior gut]** **[Posterior gut]**

Supplement: Supplemental Information 7 — Intestinal sections of Chirostoma estor were separated in the anterior and posterior gut. [file peerj-10-13052-s007.pdf]

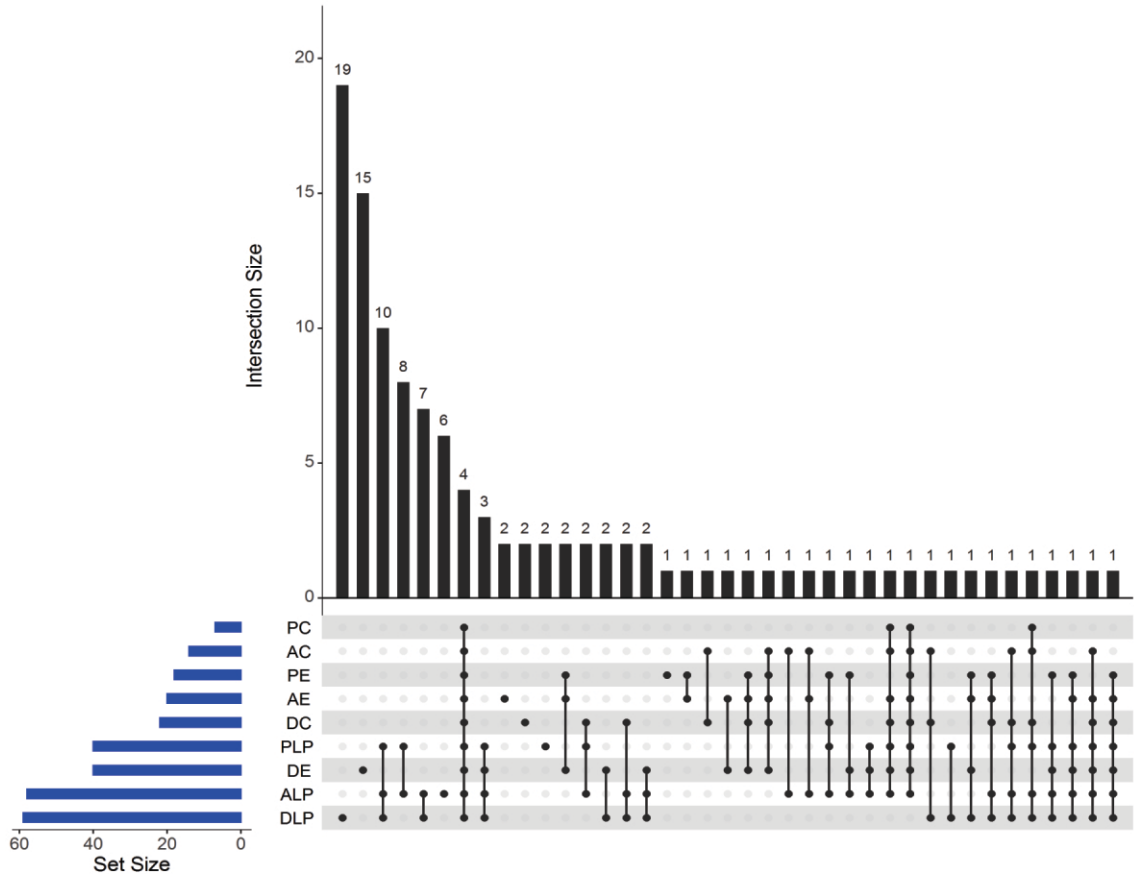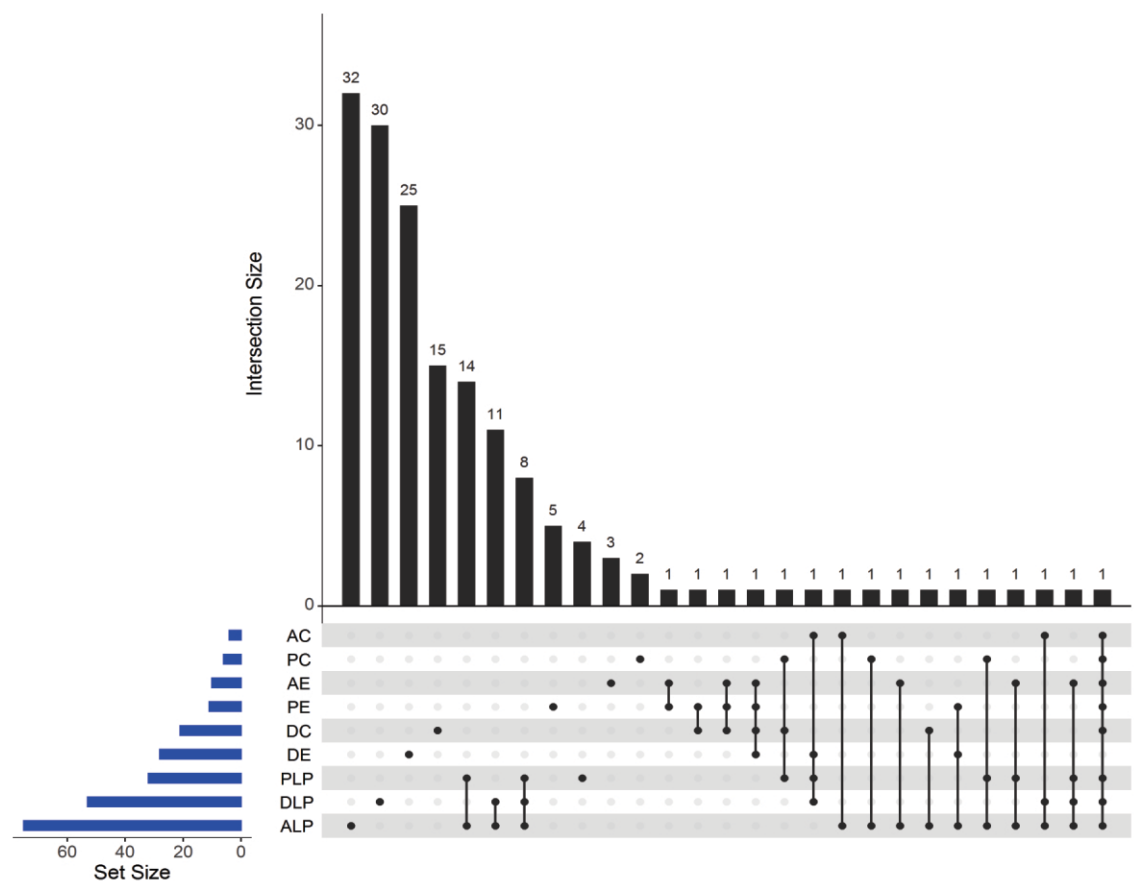

Supplement: Supplemental Information 8 — UpSet plot showing the core of microbial genera above a threshold of 80% (above) and below the threshold cutoff of 80% (below). Group set size is plotted as blue horizontal bars. Bars show the number of genera or OTUs that are unique within a specified group (in at least 80% of the samples) and dark circles with connecting bars indicate shared OTUs between multiple samples. Legends: DC, digesta of intensive culture; PC, posterior intestine of intensive culture; DLP, digesta of Lake Patzcuaro fish; PLP, posterior intestine of Lake Patzcuaro fish; ALP, anterior intestine of Lake Patzcuaro fish; AE, anterior intestine of extensive culture; PE, posterior intestine of extensive culture; DE, digesta of extensive culture; AC, anterior intestine of intensive culture. [file peerj-10-13052-s008.pdf]

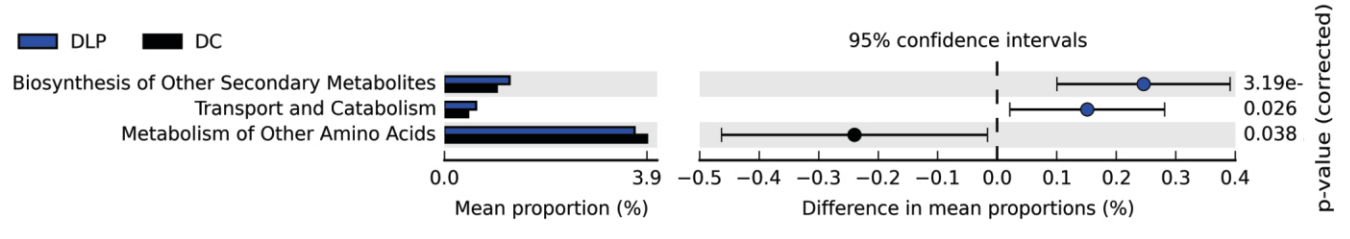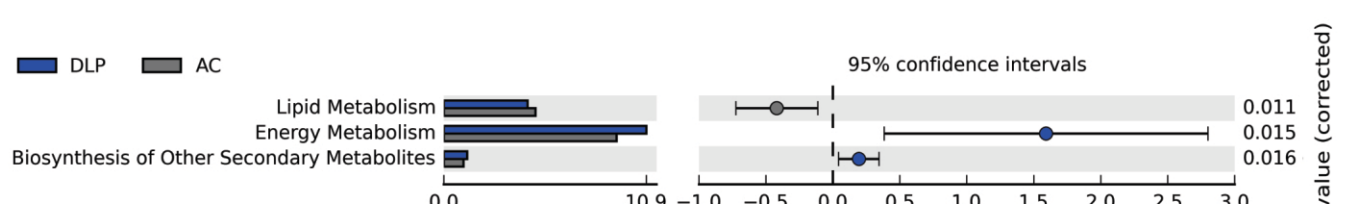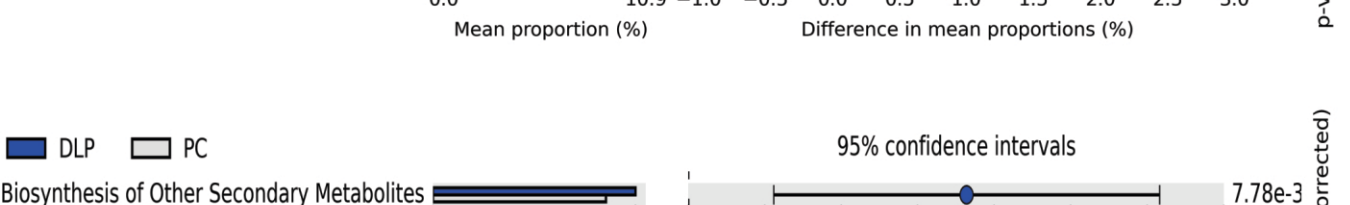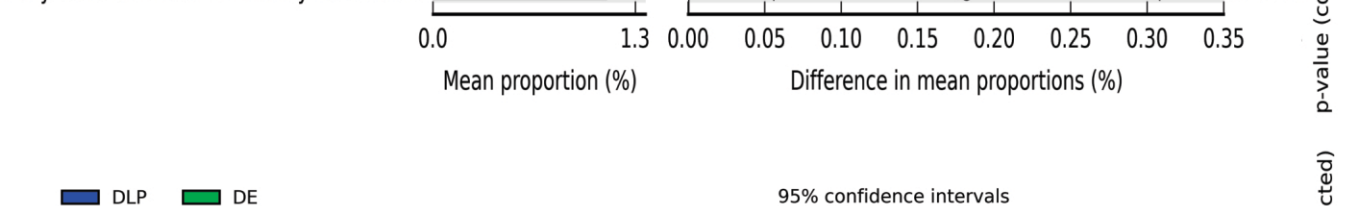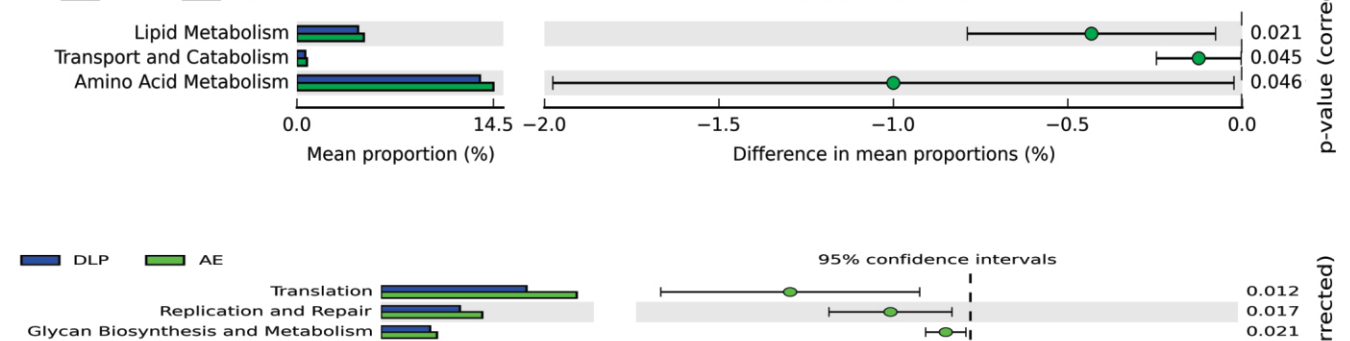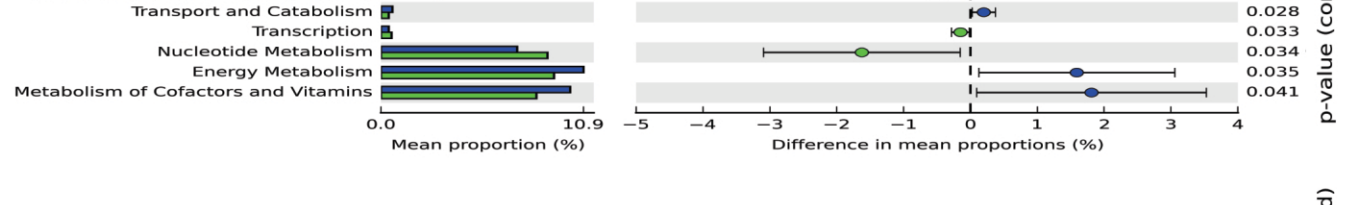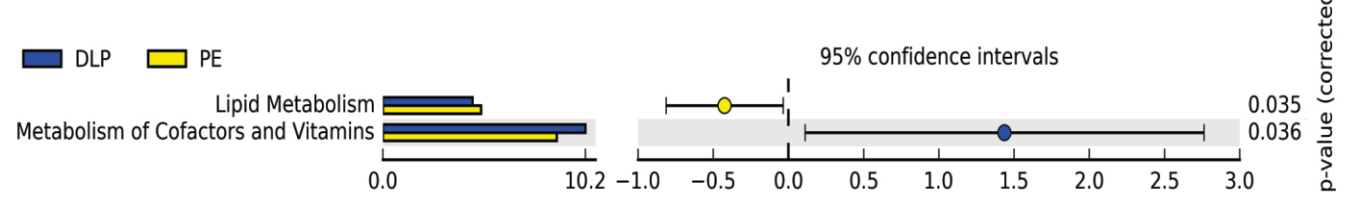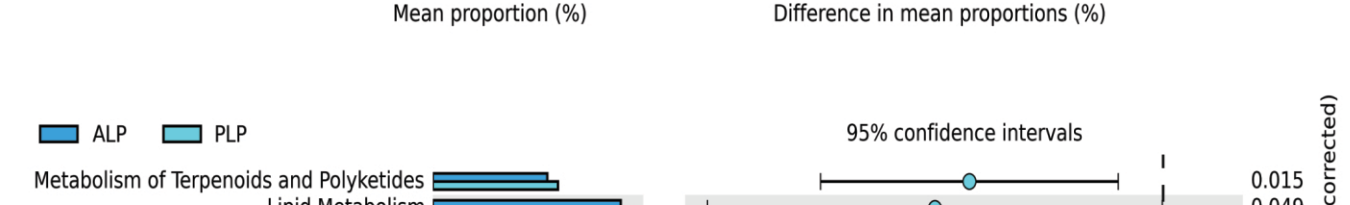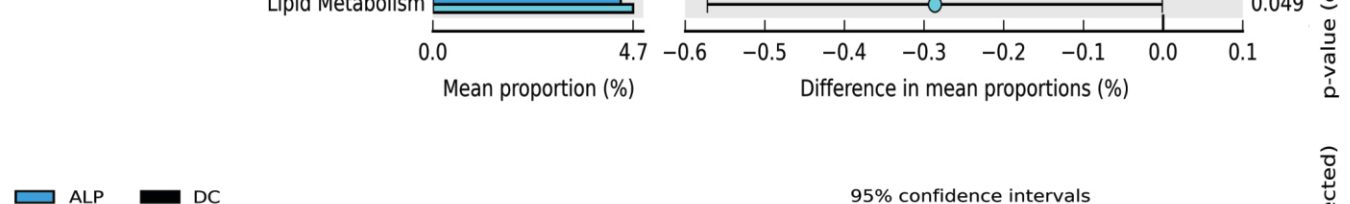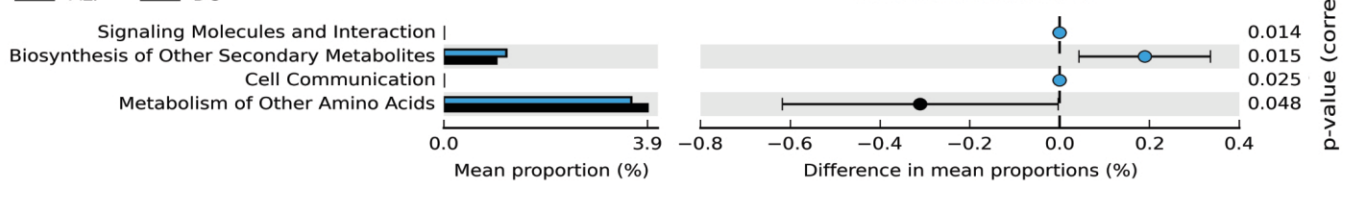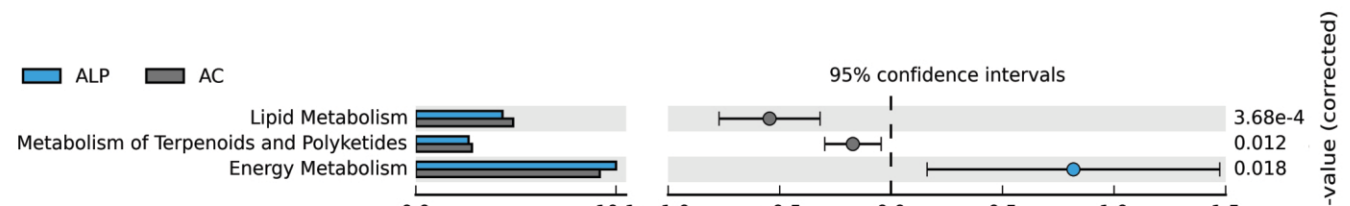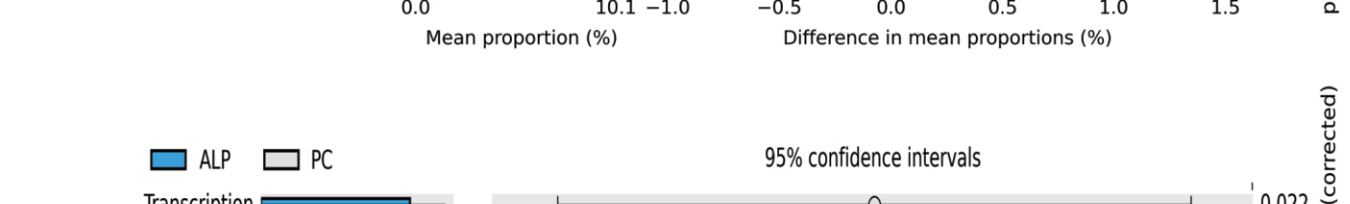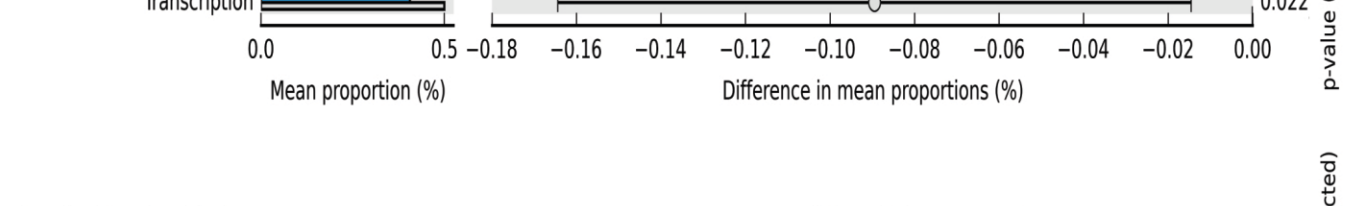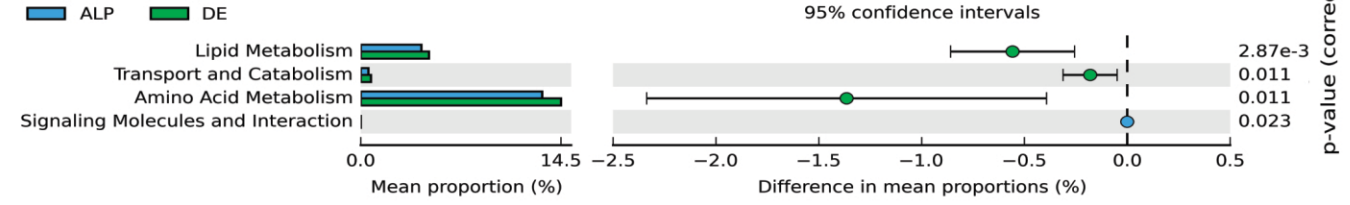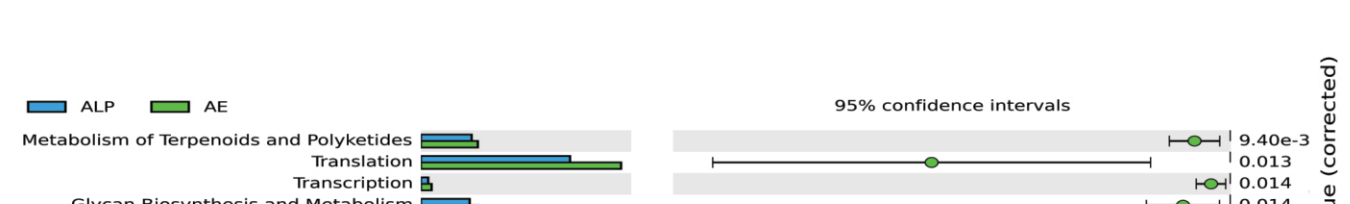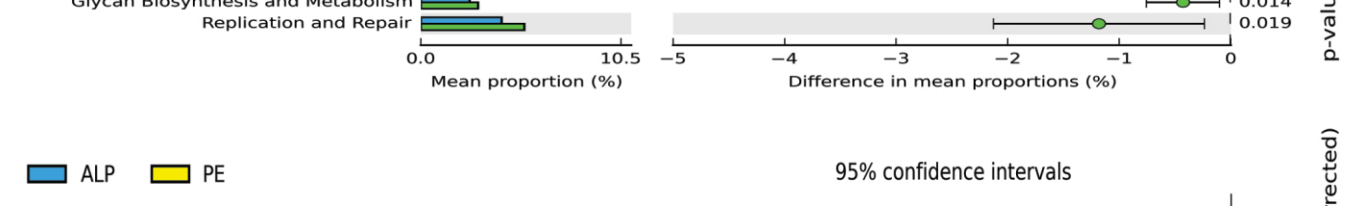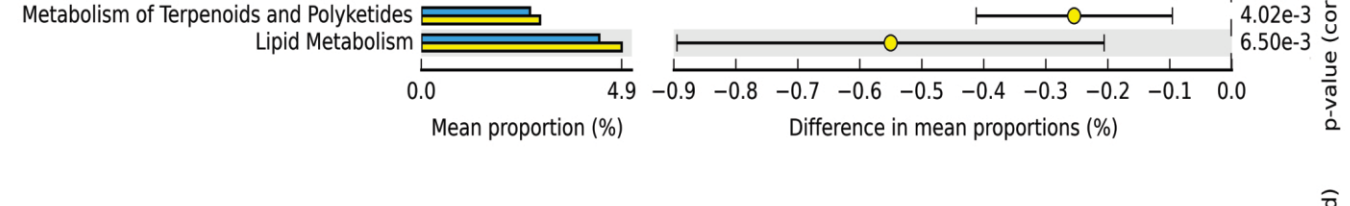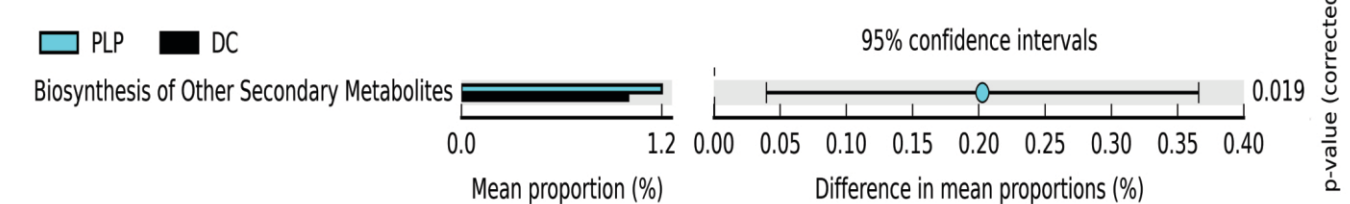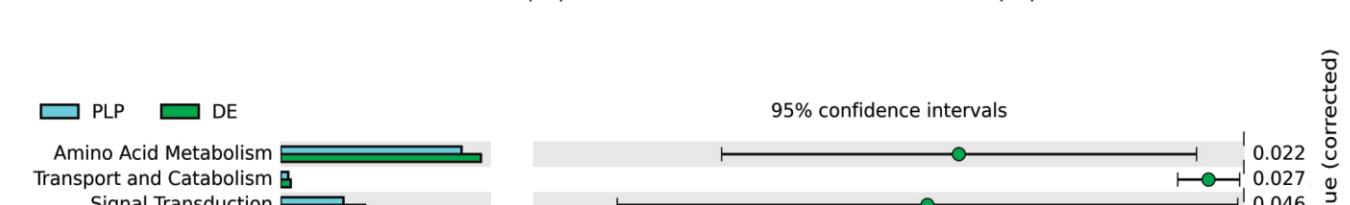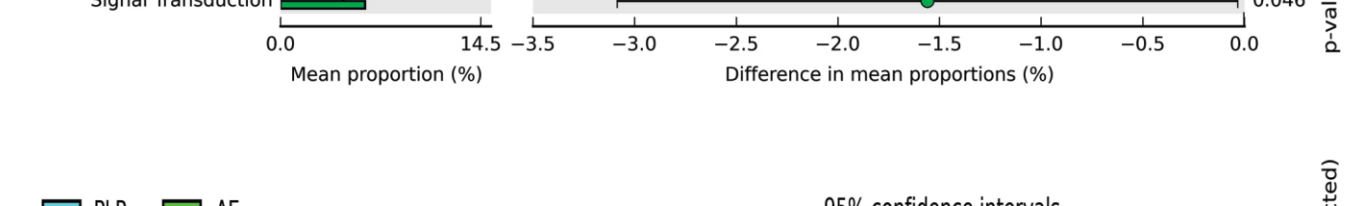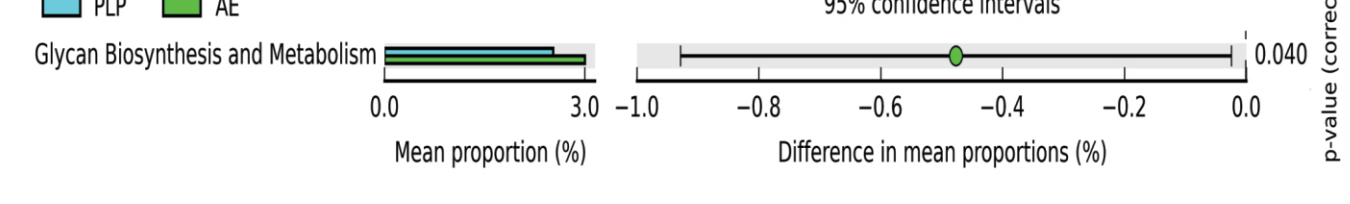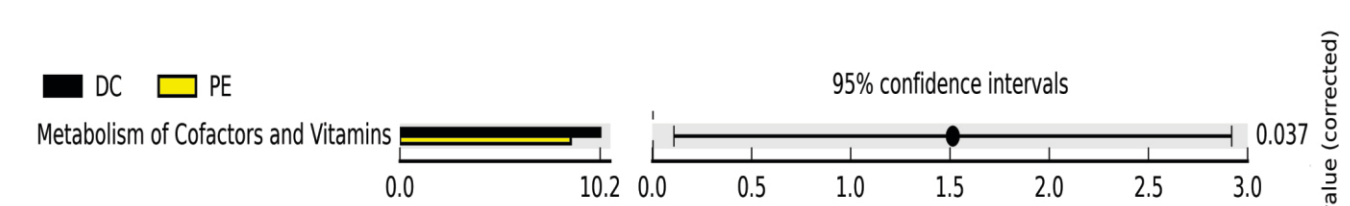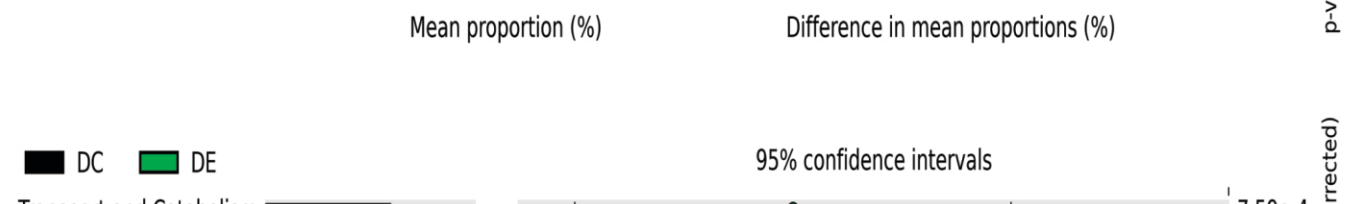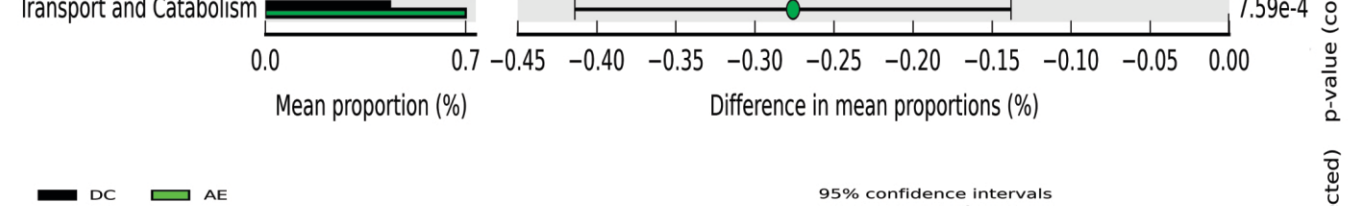

Supplement: Supplemental Information 10 — Displayed as extended error bar plots of mean proportional difference between KOs pathways of PICRUSt2 in pairwise groupings by environments or intestinal components of Chirostoma estor. Corrected p values are shown at right. The legend groups are shown: DLP, digesta/Lake Patzcuaro; ALP, anterior intestine/Lake Patzcuaro; PLP, posterior intestine/Lake Patzcuaro; DC, digesta/intensive culture; AC, anterior intestine/intensive culture; PC, posterior intestine/intensive culture; DE, digesta/extensive culture; AE, anterior intestine/extensive culture; PE, posterior intestine/extensive culture. [file peerj-10-13052-s010.pdf]
