# Supplementary material for: Loss of gut microbial diversity in the cultured, agastric fish, Mexican pike silverside (Chirostoma estor: Atherinopsidae)
Source: PeerJ. 2022 Mar 7;10:e13052. doi: 10.7717/peerj.13052 (PMC8908885; doi:10.7717/peerj.13052)
Supplement: Supplemental Information 9 — Red color represents higher value and blue color lower value. The top color bars indicate the environment and intestinal component groups. Blue bars LP, Lake Patzcuaro; black bars C, Intensive culture; green bars E, Extensive culture; cyan bars D, Digesta; brown bars A, Anterior gut; orange bars P, Posterior gut. [file peerj-10-13052-s009.pdf]

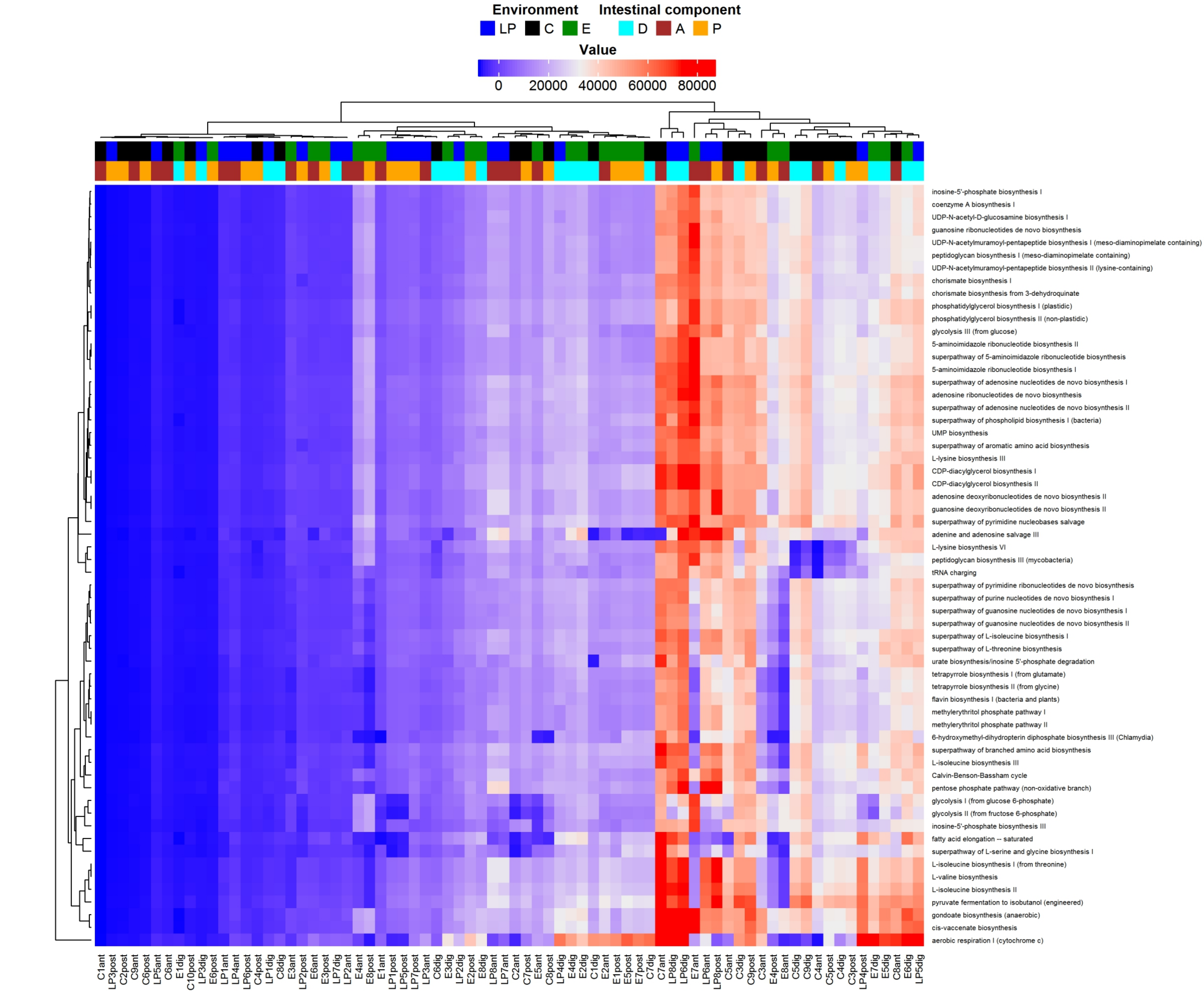
Environment
LP
C
E
Intestinal component
D
A
P
Value
0
20000
40000
60000
80000
inosine-5'-phosphate biosynthesis I
coenzyme A biosynthesis I
UDP-N-acetyl-D-glucosamine biosynthesis I
guanosine ribonucleotides de novo biosynthesis
UDP-N-acetylmuramoyl-pentapeptide biosynthesis I (meso-diaminopimelate containing)
peptidoglycan biosynthesis I (meso-diaminopimelate containing)
UDP-N-acetylmuramoyl-pentapeptide biosynthesis II (lysine-containing)
chorismate biosynthesis I
chorismate biosynthesis from 3-dehydroquinate
phosphatidylglycerol biosynthesis I (plastidic)
phosphatidylglycerol biosynthesis II (non-plastidic)
glycolysis III (from glucose)
5-aminoimidazole ribonucleotide biosynthesis II
superpathway of 5-aminoimidazole ribonucleotide biosynthesis
5-aminoimidazole ribonucleotide biosynthesis I
superpathway of adenosine nucleotides de novo biosynthesis I
adenosine ribonucleotides de novo biosynthesis
superpathway of adenosine nucleotides de novo biosynthesis II
superpathway of phospholipid biosynthesis I (bacteria)
UMP biosynthesis
superpathway of aromatic amino acid biosynthesis
L-lysine biosynthesis III
CDP-diacylglycerol biosynthesis I
CDP-diacylglycerol biosynthesis II
adenosine deoxyribonucleotides de novo biosynthesis II
guanosine deoxyribonucleotides de novo biosynthesis II
superpathway of pyrimidine nucleobases salvage
adenine and adenosine salvage III
L-lysine biosynthesis VI
peptidoglycan biosynthesis III (mycobacteria)
tRNA charging
superpathway of pyrimidine ribonucleotides de novo biosynthesis
superpathway of purine nucleotides de novo biosynthesis I
superpathway of guanosine nucleotides de novo biosynthesis I
superpathway of guanosine nucleotides de novo biosynthesis II
superpathway of L-isoleucine biosynthesis I
superpathway of L-threonine biosynthesis
urate biosynthesis/inosine 5'-phosphate degradation
tetrapyrrole biosynthesis I (from glutamate)
tetrapyrrole biosynthesis II (from glycine)
flavin biosynthesis I (bacteria and plants)
methylerythritol phosphate pathway I
methylerythritol phosphate pathway II
6-hydroxymethyl-dihydropterin diphosphate biosynthesis III (Chlamydia)
superpathway of branched amino acid biosynthesis
L-isoleucine biosynthesis III
Calvin-Benson-Bassham cycle
pentose phosphate pathway (non-oxidative branch)
glycolysis I (from glucose 6-phosphate)
glycolysis II (from fructose 6-phosphate)
inosine-5'-phosphate biosynthesis III
fatty acid elongation -- saturated
superpathway of L-serine and glycine biosynthesis I
L-isoleucine biosynthesis I (from threonine)
L-valine biosynthesis
L-isoleucine biosynthesis II
pyruvate fermentation to isobutanol (engineered)
gondoate biosynthesis (anaerobic)
cis-vaccenate biosynthesis
aerobic respiration I (cytochrome c)
C1ant LP3post C2post C9ant C6post LP5ant C6ant E1dig C10post LP3dig E6post LP1ant LP4ant LP6post C4post LP1dig C8dig E3ant LP2post E6ant E3post LP7dig LP2ant E4ant E8post E1ant LP1post LP5post LP7post LP3ant C6dig E3dig LP2dig E2post E8dig LP8ant LP7ant C2ant C7post E5ant C8post LP4dig E4dig E2dig C1dig E2ant E1post E5post E7post C7dig C7ant LP8dig LP6dig E7ant LP6ant LP8post C5ant C3dig C9post C3ant E4post E8ant C5dig C9dig C4ant C5post C4dig C3post LP4post E7dig E5dig C8ant E6dig LP5dig
